# Limited not lazy: a quasi-experimental secondary analysis of evidence quality evaluations by those who hold implausible beliefs

**DOI:** 10.1186/s41235-020-00264-z

**Published:** 2020-12-11

**Authors:** Kristy A. Martire, Bethany Growns, Agnes S. Bali, Bronte Montgomery-Farrer, Stephanie Summersby, Mariam Younan

**Affiliations:** 1 School of Psychology, The University of New South Wales, Sydney, NSW 2052 Australia; 2 School of Social and Behavioral Sciences, Arizona State University, 4701 W Thunderbird Rd, Glendale, AZ 85069 USA

**Keywords:** Conspiracy theories, Fake news, Implausible beliefs, Evidence evaluation, Decision-making, Cognitive reflection test, Forensic evidence, Analytical thinking

## Abstract

Past research suggests that an uncritical or ‘lazy’ style of evaluating evidence may play a role in the development and maintenance of implausible beliefs. We examine this possibility by using a quasi-experimental design to compare how low- and high-quality evidence is evaluated by those who do and do not endorse implausible claims. Seven studies conducted during 2019–2020 provided the data for this analysis (*N* = 746). Each of the seven primary studies presented participants with high- and/or low-quality evidence and measured implausible claim endorsement and evaluations of evidence persuasiveness (via credibility, value, and/or weight). A linear mixed-effect model was used to predict persuasiveness from the interaction between implausible claim endorsement and evidence quality. Our results showed that endorsers were significantly more persuaded by the evidence than non-endorsers, but both groups were significantly more persuaded by high-quality than low-quality evidence. The interaction between endorsement and evidence quality was not significant. These results suggest that the formation and maintenance of implausible beliefs by endorsers may result from less critical evidence evaluations rather than a failure to analyse. This is consistent with a limited rather than a lazy approach and suggests that interventions to develop analytical skill may be useful for minimising the effects of implausible claims.

## Significance statement

Information is more abundant and accessible than ever before. The constant stream of news contains true information, as well as errors, exaggeration, and lies. Consequently, some people come to believe highly implausible claims—for example, that the COVID-19 pandemic is a hoax. These beliefs can be costly for individuals and society, making it vital to understand who believes implausible claims and why. Research suggests that a ‘lazy’ uncritical style of evaluating evidence may be associated with the formation and maintenance of implausible beliefs. Our quasi-experimental study tests whether those who endorse implausible claims evaluate high-quality or low-quality evidence differently to those who do not. We argue that if those who believe implausible claims are generally ‘lazy’ uncritical thinkers, then they will find high- and low-quality evidence equally persuasive, while non-endorsers will not. Analysis of data from seven different studies shows that high-quality evidence was more persuasive overall than low-quality evidence for both endorsers and non-endorsers. However, endorsers were more persuaded by the presented evidence than non-endorsers were. These findings suggest that those who hold implausible beliefs are sensitive to evidence quality, but are more persuaded than those who do not hold implausible beliefs. Thus, implausible beliefs may result from limited evaluative skills, rather than a ‘lazy’ thinking style.

## Introduction

Information is more accessible now than ever before. The constant stream of material from news and social networks contains true information as well as errors, exaggeration, and lies. However, our capacity to process and evaluate the reliability of this information is limited and can lead to errors in thinking and judgment (Hills 2019). For example, some people come to believe highly implausible claims like conspiracy theories, fake news, and paranormal accounts. These beliefs can be costly for individuals and society (Frau-Meigs 2019; Lewandowsky et al. 2017). Indeed, we have seen that misplaced belief in fabricated, false, and implausible statements can lead to a range of undesirable behaviours like prejudice, rejection of moderate political views, disdain for scientific consensus, and a disregard for evidence-based medical advice (Allington et al. 2020; Douglas et al. 2019; Imhoff and Lamberty 2020; Zimmermann and Kohring 2020). Therefore, it is vital for us to better understand who believes implausible claims and why.

There is evidence that those who more strongly believe one implausible claim are also more likely to strongly believe other unsubstantiated claims (Bensley et al. 2020). For instance, those who endorse dubious health-related information, religious, paranormal, and conspiratorial beliefs are also more likely to be persuaded by pseudo-profound statements (a.k.a. ‘bullshit’; Pennycook et al. 2015a). Various types of implausible beliefs (e.g. magical thinking, pseudo-scientific claims, and belief in fake news) also tend to be positively correlated with each other (Barron et al. 2018; Lobato et al. 2014; Pennycook et al. 2015a; Pennycook and Rand 2019; Rizeq et al. 2020; Ståhl and van Prooijen 2018). The strength and ubiquity of these associations have led researchers to suspect that a common cognitive style may underpin many forms of implausible beliefs (Bronstein et al. 2019; Lobato et al. 2014; Rizeq et al. 2020; Ståhl and van Prooijen 2018).

### Cognitive style and implausible beliefs

A cognitive style is an individual’s preferred approach for perceiving, processing and remembering information (Zhang and Sternberg 2006). Evidence suggests a *reflexive* (or ‘Type 1’ Evans and Stanovich 2013; Kahneman 2011; Ross et al. 2016)*,* rather than a *reflective* (‘Type 2’), cognitive style is associated with the formation and maintenance of various implausible beliefs (Bronstein et al. 2019; Greene and Murphy, this issue; Pennycook et al. 2015a; Pennycook et al. 2015b; Pennycook and Rand 2020; Sindermann et al. 2020). A reflexively open-minded cognitive style describes a ‘lazy’ approach to decision-making, whereby a broad range of claims are uncritically accepted, irrespective of their epistemic value (Pennycook and Rand 2020). In contrast, a reflective cognitive style describes the tendency to more slowly analyse the information presented, question one’s intuition, and consider alternatives in decision-making (Pennycook et al. 2015b; Pennycook and Rand 2020; Zhang and Sternberg 2006).

### Examining the relationship between cognitive style and implausible beliefs

Studies that have investigated the relationship between cognitive style and implausible beliefs have generally explored this via correlations between measures of cognitive style and implausible claim endorsement. A wide variety of measures of cognitive style have been used in this literature including the Cognitive Reflection Test (Frederick 2005; e.g. Greene and Murphy this issue; Pennycook and Rand 2019; Ståhl and van Prooijen 2018), the Actively Open-Minded Thinking Scale (Stanovich and West 1997; e.g. Bronstein et. al. 2019; Rizeq et al. 2020), the Need For Cognition Scale (Cacioppo et al. 1996; e.g. Barron et al. 2018; Ross et al. 2016) and the Rational/Experiential Multimodal Inventory (Norris and Epstein 2011; e.g. Barron et al. 2018). Implausible claim endorsement has been examined using the Bullshit Receptivity Scale (Pennycook et al. 2015a; e.g. Pennycook and Rand 2019, 2020), Belief in Conspiracy Theories Inventory (Swami et al. 2010; e.g. Barron et al. 2018), Core Knowledge Confusion scale (Lindeman and Aarnio 2007; e.g. Rizeq et al. 2020), and Paranormal Belief Scale (Drinkwater et al. 2017; e.g. Ståhl and van Prooijen 2018), among others.

Overwhelmingly, these correlational studies have shown an association between cognitive style and implausible beliefs. Specifically, people who more strongly endorse implausible claims typically have more intuitive, reflexive cognitive styles (Barron et al. 2018; Greene and Murphy this issue; Lobato et al. 2014; Mikušková 2018; Pennycook et al. 2015a; Pennycook and Rand 2019, 2020; Rizeq et al. 2020; Ståhl and van Prooijen 2018). Furthermore, indicators of reflective thinking (i.e. open-mindedness and analytical thinking) have also been found to mediate the relationship between delusion-proneness, dogmatism, and fake news endorsement (Bronstein et al. 2019). These associations suggest that implausible beliefs may arise from a failure to engage in a deliberative evaluation of relevant information—resulting in a failure to identify the weaknesses and implausibility of epistemically suspect claims. However, other possibilities may explain the association between cognitive style and implausible belief endorsement.

The Motivated System 2 Reasoning (MS2R) account is one alternative explanation, which suggests that deliberation may actually bias people to favour information that aligns with their ideology—irrespective of epistemic value (Pennycook and Rand 2020). That is, a reflective cognitive style might *increase* belief in implausible claims that are consistent with one's own perspective via effortful deliberation. Pennycook and Rand (2019) tested the MS2R account by examining the relationship between cognitive style and belief in ideologically in/consistent (i.e. partisan) real or fake news. However, they found that those with a more reflective analytical style were *better* at discerning between real and fake news—irrespective of ideological consistency. This result led to the view that people may endorse implausible claims because they are ‘lazy, not biased’ evidence evaluators (Pennycook and Rand 2019). This interpretation is also supported by the results of experimental studies.

In a series of experiments, Swami et al. (2014) found that interventions that create cognitive disfluency and slow down information processing significantly reduce the endorsement of conspiracy claims. Similarly, Bago et al. (2020) found that participants believe false headlines more when evaluating under time pressure and cognitive load than when given unlimited time to assess the claims. Taken together, this evidence suggests that promoting reflective analysis can improve evidence evaluations and reduce the endorsement of implausible claims. However, researchers have not yet examined whether those who endorse implausible claims actually analyse evidence more poorly, or differently, than those who do not.

Researchers have also not examined whether errors in evaluating brief pseudo-profound statements or news headlines (e.g. Bago et al. 2020; Bronstein et al. 2019; Pennycook et al. 2015a; Pennycook and Rand 2019, 2020) generalise to the evaluation of more realistic materials like news articles, interviews, blogs, or opinion pieces. The tasks used in the previous research generally contain little if any substantive content beyond a statement, a headline, or a few lines of text. For example, even extended and reflective consideration of the fake news headline ‘*Trump on Revamping the Military: We’re Bringing Back the Draft*’ (Pennycook and Rand 2020) does not easily reveal the objective truth of that claim. Indeed, materials like this contain few cues that can be relied upon to differentiate between the true and fake claims aside from plausibility.

Thus, participants in the previous research have been given limited *scope* to engage in a reflective analysis—even if they wanted to. This leaves open the possibility that something other than reflective analysis separates good from poor performance on these evidence evaluation tasks and suggests it is important to provide decision-makers with more sophisticated tests of their analytical ability (Ståhl and van Prooijen 2018). For example, by presenting rich sources of information that contain objective strengths and weaknesses relevant to the reliability of the claims. One source of this type of information is expert evidence presented in courts.

### Evidence quality evaluation in forensic contexts

Lay jurors in civil and criminal trials are routinely presented with complex technical and scientific information by expert witnesses (Gross 1991; Hilbert 2019; Jurs 2015). It is their duty to determine the outcome of a case based on a rational assessment of the evidence presented to the court (Edmond 2015; Raeder 2003; Thayer 1890). Jurors are directed by the judge to evaluate the evidence and decide which claims are sufficiently credible for belief (e.g. Eleventh Circuit Pattern Jury Instructions, criminal 2020; Judicial Commission of New South Wales 2020; for discussions, see Brewer 1998; Edmond 2015; Ward 2017). Yet, as in other contexts, jurors sometimes make mistakes about information quality and veracity (McAuliff and Duckworth 2010; McAuliff et al. 2009). These mistakes can be highly consequential, resulting in innocent people being convicted (or held liable) and punished for offences they did not commit (Derwin 2018; Garrett 2017; Garrett and Neufeld 2009).

Scholars and authoritative scientific bodies have raised concerns about the quality of expert evidence for decades (Giannelli 1993; Hand 1901; Hilbert 2019; Mnookin 2007; National Research Council of the Academies of Science [NRC] 2009; President’s Council of Advisors on Science and Technology [PCAST] 2016). These concerns primarily relate to genuinely held opinions that are plausible, but ultimately incorrect or insufficiently reliable. For example, low-quality opinions are those that are given without sufficient evidence that the underpinning science is repeatable, reproducible, or accurate (PCAST 2016); that is expressed incorrectly or without appropriate qualification (NRC 2009), where the proficiency of the examiner has not been demonstrated (Garrett and Mitchell 2018; Martire and Edmond 2016) and where biasing contextual information has not been appropriately disclosed or managed (Dror 2016; NRC 2009). Conversely, high(er)-quality opinions are those based on foundationally valid methods and techniques, that are expressed using valid terminology, and that appropriately disclose assumptions and limitations (NRC 2009). These opinions are produced by practitioners with appropriate qualifications, demonstrated skill, and who have limited, declared, or removed potentially biasing influences (Edmond et al. 2016; Martire et al. 2020). The forensic context, therefore, provides a novel—yet realistic setting—for examining possible differences in evidence quality evaluations between those who do and do not endorse implausible claims.

## The present study

In this paper, we conduct a quasi-experimental secondary analysis of data from seven studies to examine whether those who hold implausible beliefs evaluate objectively higher- or lower-quality forensic evidence differently to those who do not hold implausible beliefs. If, as past analysis suggests, those who endorse implausible claims have a ‘lazy’, reflexive cognitive style and do not engage in analysis of the evidence, we would expect endorsers to be equally persuaded by low- and high-quality evidence because their uncritical approach leads them to be insensitive to epistemic value (Pennycook and Rand 2019).

However, if those who hold implausible beliefs do engage in some—albeit imperfect—analysis, then we would anticipate some sensitivity to evidence quality whereby high-quality evidence is more persuasive than low-quality evidence. If endorsers complete this evaluation differently to non-endorsers—as we might anticipate given that one group is persuaded by highly improbable claims and the other is not—then we might also expect an interaction between evidence quality and endorsement status. This interaction could involve over belief of low-quality evidence and/or under belief of high-quality evidence by endorsers compared to non-endorsers.

## Method

### Data and design

We report a secondary analysis of data collected from seven studies conducted by members of a forensic decision-making research group. Each of the seven primary studies was originally designed to examine the effects of various aspects of evidence quality on perceptions of evidence persuasiveness (i.e. credibility, value, and/or weight; see Table 1 for an overview). Although it was not the main aim of these studies, our research group was also interested to know whether people who believe implausible claims generally evaluate evidence differently to those who do not. To examine this phenomenon, we measured implausible claim endorsement in each study. It is this data that we analyse here using a 2 (evidence quality: high vs. low) × 2 (implausible claim endorsement: endorser vs. non-endorser) between-subjects quasi-experimental design.

**Table 1:** Summary of primary studies contributing to preregistered quasi-experimental secondary analysis

Primary study	*N*	Sample	Design	Factor 1 (Levels)	Factor 2 (Levels)	Factor and level included in preregistered secondary analysis	
						High quality	Low quality
1	106	Mturk	Factorial	Attractiveness (Absent, High, Low)	Expert persuasion expectancy (a.k.a.‘ExPEx’ Strong, Weak)	Attractiveness Absent/Strong ExPEx	Attractiveness Absent/Weak ExPEx
2	116	Mturk	Factorial	Attractiveness (Absent, High, Low)	Expert persuasion expectancy (a.k.a. ‘ExPEx’ Strong, Weak)	Attractiveness Absent/Strong ExPEx	Attractiveness Absent/Weak ExPEx
3	54	Mturk	Oneway	Legal admissibility (Control, Explicit Admit, Implicit Admit, Explicit Exclude)	-	Explicit Admit	Explicit Exclude
4	96	Mturk	Factorial	Legal admissibility (Explicit Admit, Implicit Admit, Explicit Exclude)	Expert ability (High, Low)	Explicit Admit/High ability	Explicit Exclude/Low ability
5	138	Mturk	Factorial	Discipline reliability (High, Low)	Report disclosure (Detailed, sparse)	High reliability/Detailed disclosure	Low reliability/Detailed disclosure
6	326	Mturk	Oneway	Reasoning measure (versions 1, 2, 3)	–	–	Reasoning measure versions 1, 2 and 3^a^
7	37	Student	Factorial	Analysis method (Biased, Unbiased)	Method disclosure (Present, Absent)	Unbiased analysis /Disclosure Present	Biased analysis/Disclosure Absent

^a^All participants in Study 6 evaluated a low-quality (flawed) expert report prior to receiving the experimental manipulation

Evidence quality was varied in this study by a priori selecting one relatively high-quality and one relatively low-quality evidence condition from the seven primary studies (see Table 1). One high- and one low-quality condition was selected for analysis from each primary study except Study 6, where all three conditions involved low-quality evidence. When combined, these 15 conditions produced an evidence quality manipulation that varied aspects of scientific rigour and transparency, methodological reliability, source trustworthiness, expert proficiency, and legal admissibility. The details of each manipulation are reported in the ‘Evidence Quality’ section below.

Implausible claim endorsement was determined by responses to implausible claims about vaccines, global warming, and a flat earth. ‘Endorsers’ were participants who rated one or more of the three claims greater than or equal to 75 on a scale from 0 ‘not at all’ to 100 ‘definitely true’. Non-endorsers were those who rated all three claims lower than 50. The dependent variables were ratings of evidence credibility, value, and weight (i.e. ‘persuasiveness’) from 0 to 100.

This design, including the data for in/exclusion, high-/low-quality conditions, non-/endorsement criteria, and analytic approach, was preregistered before formal or informal inspection of implausible claim items, computation of endorsement status, or examination of the effects of endorsement status and evidence quality on the dependent variables (AsPredicted #40589; https://aspredicted.org/3rv9g.pdf).

### Participants

Of the original 1,747 eligible participants in 33 conditions from the seven primary studies, 873 participants in 15 conditions were selected for inclusion in the secondary analysis a priori. All participants were based in the USA, reported they were jury-eligible, completed the study online, and were recruited between June 2019 and May 2020. Participants from Studies 1–6 were recruited online through Amazon Mechanical Turk, had approval ratings > 95% for their past work, and were compensated up to US$10 per hour (*n* = 836). Participants from Study 7 were students recruited from a large south-western university in the USA who received course credit for their participation (*n* = 37). All participants completed a reCAPTCHA to ensure respondents were human (von Ahn et al. 2008). The combined sample contained 125 ‘endorsers’ (14.3%) and 621 ‘non-endorsers’ (71.7%). We excluded the 127 participants who did not fit our preregistered endorsement inclusion criteria (i.e. those who rated all three implausible claims between 50 and 75). After exclusions, data from 746 participants were retained for analysis. See Table 2 for demographic information. The majority of this sample identified as male (55.2%), and the mean age was 37.2 years (SD = 11.7; range = 18–74). The majority identified as White/Caucasian (76.3%), and 53.8% reported college/university as their highest level of education.

**Table 2:** Participant demographic information

Factor	Total sample	Study							‘Endorsers’	‘Non-endorsers’
		1	2	3	4	5	6	7		
Endorsement										
*N*	873	106	116	54	96	138	326	37	–	–
% ‘Endorsers’	14.3	15.1	11.2	11.1	20.8	9.4	15.3	18.9	–	–
% ‘Non-endorsers’	71.1	75.5	76.7	79.6	63.5	71.7	69.3	62.2	–	–
Age										
*n*	746	96	102	49	81	112	276	30	125	621
Mean	37.2	37.0	36.0	36.6	37.8	35.0	40.3	21.3	40.1	36.7
SD	11.7	11.6	10.5	10.2	12.0	8.9	12.3	3.4	12.8	11.4
Gender										
*n*	746	96	102	49	81	112	276	30	125	621
% Female	44.1	49.0	51.0	40.8	43.2	37.5	40.6	70.0	42.4	44.4
% Male	55.2	51.0	47.1	59.2	56.8	62.5	59.1	23.3	57.6	54.8
% Gender diverse	0.7	0.0	2.0	0.0	0.0	0.0	0.4	6.7	0.0	0.8
Highest level of education										
*n*	716	96	102	49	81	112	276	0	118	598
% Less than high/secondary	0.4	0.0	2.0	0.0	0.0	0.0	0.4	–	0.0	0.5
% High/secondary school	23.5	25.0	20.6	22.4	19.8	28.6	23.2	–	26.3	22.9
% Trade qualification	6.1	2.1	2.0	4.1	9.9	9.8	6.9	–	11.9	5.0
% College/university	53.8	57.3	51.0	63.3	49.4	51.8	54.0	–	44.9	55.5
% Masters degree	13.4	11.5	22.5	8.2	16.0	8.9	12.7	–	13.6	13.4
% Doctoral degree	0.8	3.1	1.0	2.0	0.0	0.0	0.4	–	0.8	0.8
% Professional qualification	2.0	1.0	1.0	0.0	4.9	0.9	2.5	–	2.5	1.8
Ethnic/cultural identity										
*n*	746	96	102	49	81	112	276	30	125	621
% White/Caucasian	76.3	79.2	80.4	69.4	72.8	71.4	80.8	50.0	79.2	75.7
% African-American	9.1	5.2	7.8	6.1	11.1	9.8	10.9	6.7	10.4	8.9
% Hispanic	5.4	2.1	4.9	6.1	7.4	4.5	2.9	36.7	4.8	5.5
% Asian	7.0	10.4	4.9	16.3	6.2	10.7	4.3	0.0	2.4	7.9
% Native American	0.8	2.1	1.0	2.0	0.0	1.8	0.0	0.0	1.6	0.6
% Pacific Islander	0.3	0.0	0.0	0.0	1.2	0.0	0.0	3.3	0.0	0.3
% Other	1.2	1.0	1.0	0.0	1.2	1.8	1.1	3.3	1.6	1.1

### Materials

#### Evidence quality

Evidence quality varied in different ways in each of the primary studies. In this section, we report the original research question for each primary study, a summary of the experimental manipulations, and a description of the high- and/or low-quality evidence included in this secondary analysis. Detailed descriptions of all manipulations, measures, and procedures for each primary study are also available at https://tinyurl.com/y4e75wo2.

##### Study 1

Examined the effect of expert attractiveness and expert quality on perceptions of evidence persuasiveness (preregistered at https://tinyurl.com/y2h46ddy). Attractiveness (absent, high, low) was varied using images of two male experts, one rated as high in attractiveness and the other rated as low in attractiveness. Expert quality was varied by constructing a forensic gait expert who was either ‘strong’ or ‘weak’ on each of the eight attributes in the Expert Persuasion Expectancy (ExPEx) framework (i.e. foundation, field, speciality, ability, opinion, support, consistency, and trustworthiness; see Martire et al. 2020). The strong-ExPEx/attractiveness-absent condition served as high-quality evidence for the secondary analysis. Participants in this condition read about a validated technique, used by a practitioner with general and specifically relevant qualifications, who was unbiased and provided a strong opinion that other experts independently verified. The weak-ExPEx/attractiveness-absent condition served as low-quality evidence for the secondary analysis. Participants in this condition read about an invalid technique, used by a practitioner with irrelevant general and specialist qualifications, who was partisan and unsure about their opinion. Other experts also disagreed with the opinion presented.

##### Study 2

Had the same primary aim and attractiveness manipulation as Study 1. However, in this study participants evaluated a lengthy (15-page) trial transcript adapted from the real testimony of an expert witness providing speech spectrography evidence (preregistered at https://tinyurl.com/y4glmued). Expert quality was again varied from ‘strong’ to ‘weak’ using the ExPEx framework. The strong-ExPEx/attractiveness-absent condition served as high-quality evidence for the secondary analysis. Participants in this condition read about a valid technique, used by a practitioner with relevant qualifications and extensive specialist training, who employed bias mitigation strategies, used a valid form of expression, and whose work was independently verified and agreed with by two other experts. The weak-ExPEx/attractiveness-absent condition served as low-quality evidence for the secondary analysis. Participants in this condition read about an unvalidated technique, used by a practitioner who trained in an irrelevant field, who had limited specialist training or experience, who was ignorant of and displayed bias, who provided invalid opinions, and whose work was not independently reviewed or verified by relevant experts.

##### Study 3

Examined the impact of judicial admissibility decisions on evidence persuasiveness. Participants evaluated a brief description of a bicycle helmet product evaluation provided by an engineering professor (see Schweitzer and Saks 2009). There were four types of judicial admissibility decision: control, implicit-admit, explicit-admit, and explicit-exclude. Those in the control condition were given no legal context for their evaluations of the professors’ product evaluation. Those in the implicit-admit condition were told they were making their judgements in the context of a civil liability trial but were not given information about evidence admissibility. Those in the explicit-admit condition were told that the professors’ evidence was subject to a thorough judicial review and was admissible for their consideration (i.e. could be relied upon in their decision-making). This condition served as high-quality evidence for the secondary analysis. Participants in the explicit-exclude condition were told that after a thorough judicial review the evidence was not admitted (i.e. should not be relied upon in their decision-making). This condition served as low-quality evidence for the secondary analysis.

##### Study 4

Examined the effects of expert ability and judicial admissibility decisions on evidence persuasiveness (preregistered at https://tinyurl.com/yxfbfs5e). There were three types of judicial admissibility decision in this study: control, explicit-admit, explicit-exclude. These conditions were operationalised the same way as in Study 3. The experimental materials also included information about ‘high’ or ‘low’ expert ability. In the high-ability conditions, participants were told that the engineering professor providing evidence had scored 90% accuracy on relevant proficiency tests. In the low-ability conditions, participants were told that the engineering professor providing evidence had scored 50% accuracy on relevant proficiency tests. The high-ability/explicit-admit condition served as high-quality evidence for the secondary analysis. The low-ability/explicit-exclude condition served as low-quality evidence in the secondary analysis.

##### Study 5

Examined the effects of discipline reliability and level of disclosure on evidence persuasiveness (preregistered at https://tinyurl.com/yyjsvzad). Participants read a report either about a high-reliability (fingerprint analysis) or low-reliability forensic discipline (footwear analysis). The report provided either a detailed- or a sparse-disclosure of important information about the evidence and opinion. In the detailed disclosure conditions, the report was modelled on best-practice recommendations for expert reports submitted to police and courts (per Edmond et al. 2016). In the sparse-disclosure conditions, important information was omitted. The high-reliability/detailed-disclosure condition served as high-quality evidence in the secondary analysis. In this condition, participants read a detailed fingerprint analysis report stating that: studies show fingerprint experts have expertise but can still make errors; the error rates for the discipline could be as high as 1 in 306 or 1 in 18 and that no forensic method other than nuclear DNA had been shown to demonstrate a connection between evidence and an individual or source. The low-reliability/detailed-disclosure condition served as low-quality evidence in the secondary analysis. Participants in this condition read a detailed footwear analysis report, indicating that no studies have looked at error rates for footwear evidence, or examined whether footwear experts possess genuine expertise. They were also told that no appropriate black-box studies have supported the foundational validity of footwear analysis.

##### Study 6

Examined how different reasoning measures predict evidence persuasiveness (preregistered at https://tinyurl.com/yyp2dm3m). Participants in this study read and evaluated the same detailed expert footwear comparison report before completing one of three different measures to assess their reasoning. The report contained three important flaws that undermined the quality of the evidence. Specifically, the report contained information that the expert performs with 45–55% accuracy on relevant proficiency tests, a fallacy in the reporting of the results (i.e. a prosecutors’ fallacy; Thompson and Schumann 1987) and limitations to the quality of the footwear impression images used in the analysis. In all three conditions, participants evaluated the same low-quality evidence—only the dependent measures differed by condition. As such, the data from this study add to the data for *low*-quality evidence in our analyses and only speak to evidence-quality *differences* when combined with the data from the other six primary studies.

##### Study 7

Examined the effects of analysis method and method disclosure on evidence persuasiveness (preregistered at https://tinyurl.com/yyp2dm3m). Participants in this study read an opinion from a DNA analyst stating either the ‘biased’ (race-specific) or ‘unbiased’ (race-neutral) assumptions associated with the analytic method. Analyses completed using race-specific rather than race-neutral DNA databases are often conducted to produce more conservative random match probability estimates that inflate the likelihood that the defendant was the source of DNA associated with a crime (Oldt and Kanthaswamy 2020). Participants were also either given an additional statement explicitly disclosing the method used (race-specific or race-neutral database) or were provided with no explicit information about the method. The unbiased-method/disclosure-present condition served as high-quality evidence for the secondary analysis. Participants in this condition read a statement from the DNA analyst that the probability of observing the match between the suspect and crime scene samples was 100 million times greater than the probability of observing the same match ‘*assuming that someone else, regardless of race, was the contributor*’. They were then also told that this estimate was calculated ‘*from a database that includes DNA frequency data from individuals of all races*’. The biased-method/disclosure-absent condition served as low-quality evidence for the secondary analysis. Participants in this condition read a statement from the DNA analyst ‘*assuming that someone else of the same race was the contributor*’. These participants were not explicitly informed that the analysis was completed using a race-specific database.

#### Evidence evaluations

Participants in Studies 1–5 and 7 answered three questions about the specific type of evidence they were presented using on-screen sliders: (1) *How credible was the expert?* From 0 ‘not at all’ to 100 ‘definitely credible’; (2) *How valuable was the evidence?* From 0 ‘not at all’ to 100 ‘definitely valuable’; (3) *How much weight do you give to the evidence?* From 0 ‘none at all’ to 100 ‘the most possible’. Participants in Study 6 only answered question three.

#### Implausible claim endorsement

To minimise social desirability in responding, the three implausible claims were randomly interspersed throughout an 11-item general knowledge battery. Participants rated general knowledge statements (e.g. *Sharks are mammals* and *A kilogram is heavier than a gram*) from 0 ‘not at all’ to 100 ‘definitely true’. Two of the three implausible claims included in the battery were based on items used in past research: *Vaccines are harmful, and this fact is covered up* (Jolley and Douglas 2014), and *Global warming is a hoax* (van der Linden 2015). The third item was new: *The earth is flat.* Implausible claim ‘endorsers’ demonstrated a high degree of belief in an implausible claim by rating at least one of these three items ≥ 75 out of 100 for truth. ‘Non-endorsers’ rated all three items lower than 50, indicating they regarded all the implausible claims more false than true. Data from participants that rated these items between 50 and 75 were excluded from the analysis. Ratings were provided using an on-screen slider which had to be moved to progress in the study.

### Procedure

After providing consent, participants were presented the evidence materials containing relevant quality information for their study and condition. They then answered study-specific questions about their perceptions of the evidence and completed the evidence persuasiveness measures (i.e. credibility, value, and/or weight). The general knowledge battery containing the implausible claims was presented after all study-specific dependent measures and before the demographic questions (except in studies 2 and 5, where it followed the demographic questions). Finally, all participants were debriefed and thanked for their participation.

### Analysis

Our analysis plan was preregistered. The R *lmer* (*v. 1.1-25*; Bates et al. 2015) and *lmerTest* (*v. 3.1-3*; Kuznetsova et al. 2017) packages were used to construct a linear mixed-effects model predicting ‘persuasiveness’ (i.e. credibility, value, and weight) from the interaction between evidence quality (low or high) and endorsement status (endorser or non-endorser). A random effect was included for each participant nested in each study. This allowed participant ratings of persuasiveness to vary between studies or participants within each study. The *lme.dscore* function from the *EMAtools* package (*v. 0.3.1*; Kleiman 2017) was used to calculate effect sizes for the fixed effects in the model.

## Results

### Implausible claim endorsement

The global warming claim was rated 75 or higher (i.e. endorsed) by 85 participants (11.4%), the vaccine claim was endorsed by 44 participants (5.9%), and the flat earth claim was endorsed by 19 participants (2.5%). See “Appendix A” for the distribution of responses for each implausible claim by endorsement status. Most participants (83.9%) rated no implausible claims over 75, 13.1% endorsed one claim, 2.1% endorsed two claims, and 0.8% endorsed all three implausible claims.

### Evidence evaluations

**Fig. 1:**
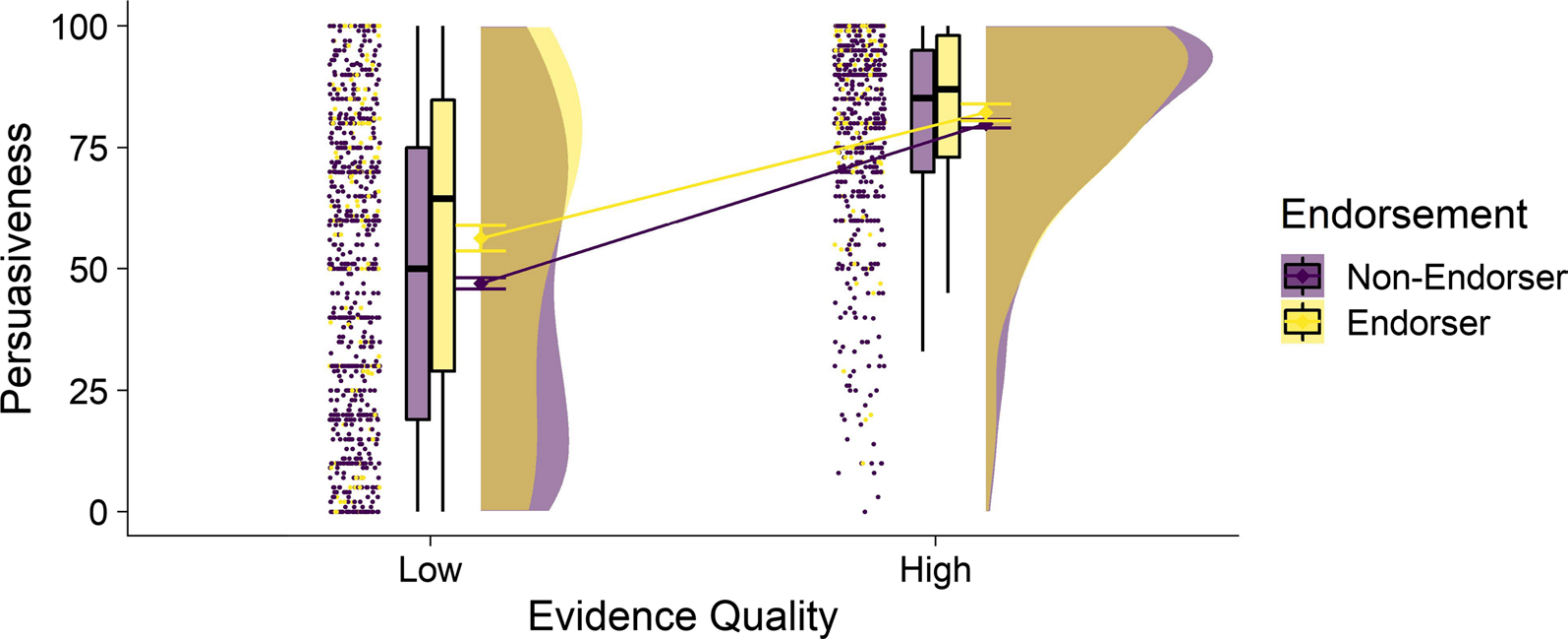
Persuasiveness Ratings for Endorsers and Non-Endorsers by Evidence Quality (*N* = 746). *Note:* Raincloud plots depict from left to right: (1) raw jittered data points; (2) Box-and-Whisker plots with median (middle bar), first and third quartiles (boxes either side of bar) and no further than 1.5 × the interquartile range (whiskers); (3) means (diamonds) and 95% confidence intervals (error bars);(4) distributions showing the frequency of scores

Overall, participants were significantly more persuaded by high-quality (*M* = 80.3, SD = 20.4) than low-quality evidence (*M* = 48.5, SD = 32.2; *b* = 32.59, SE = 2.42, *t*_(673.53)_ = 13.45, *p* < 0.001, 95% CI [27.87, 37.35], Cohen’s *d* = 1.04; see Fig. 1). Endorsers were also significantly more persuaded by the presented evidence (*M* = 67.3, SD = 30.9) than non-endorsers (*M* = 61.4, SD = 31.9; *b* = 10.21, SE = 3.06, *t*_(775.86)_ = 3.33, *p* < 0.001, 95% CI [4.21, 16.21], *d* = 0.24). The interaction between endorsement and evidence quality was not significant (*b* = − 9.22, SE = 5.31, *t*_(700.75)_ = 1.74, *p* = 0.083, 95% CI [− 19.61, 1.21], *d* = 0.13), but it is important to note that this result does not constitute evidence against such an interaction. Endorsers’ ratings of low-quality (*M* = 56.3, SD = 33.4) and high-quality evidence (*M* = 82.2, SD = 19.0) did not significantly differ from non-endorsers ratings of low-quality (*M* = 47.0, SD = 31.7) and high-quality evidence (*M* = 79.9, SD = 20.6). See “Appendix B” for figures showing persuasiveness by evidence quality and endorsement status within each study. See “Appendix C” for post hoc analyses using all eligible participants from the primary studies (*N* = 1,747) and different definitions of non-/endorsement status.

## Discussion

In this study, we examined whether people who endorse implausible claims evaluate high- or low-quality evidence differently to people who do not. We found both similarities and differences in how endorsers and non-endorsers assigned credibility, value, and weight to forensic evidence. Compared to non-endorsers, endorsers were more persuaded by the evidence they were presented. However, both endorsers and non-endorsers were more persuaded by high-quality than low-quality evidence. These results are inconsistent with predictions based on previous correlational research and suggest new avenues for interventions to reduce the harms associated with implausible claim endorsement.

In terms of similarities, we found that high-quality evidence was valued more than low-quality evidence, irrespective of whether or not a person held a strong belief that vaccines are harmful, the earth is flat, or that global warming is a hoax. That is, compared to non-endorsers, endorsers did not significantly differ in their sensitivity to our manipulations of expert characteristics such as legal relevance, trustworthiness, proficiency, methodological rigour, reliability, and transparency. Although past research suggests such evaluations may be far from optimal (McAuliff and Duckworth 2010; McAuliff et al., 2009), the observed similarity between endorsers and non-endorsers is not what we expected based on previous research.

Past studies have shown that people who more strongly endorse implausible claims typically have a more intuitive, reflexive cognitive style (Barron et al. 2018; Lobato et al. 2014; Mikušková 2018; Pennycook et al. 2015a; Pennycook and Rand 2020; Rizeq et al. 2020; Ståhl and van Prooijen 2018). As a result, researchers have inferred that people endorse implausible claims because they are lazy and ‘fail to think’ (Pennycook and Rand 2019, p. 47). This led us to predict that if people who endorse implausible claims do not analyse, then they would be equally persuaded by high-quality and low-quality evidence. However, that is not what we found.

Our results suggest that endorsers and non-endorsers both completed some form of reflective analysis when given the opportunity to evaluate claims with a diverse array of strengths and weaknesses. This result is consistent with Greene and Murphy’s finding (this issue) that levels of analytical reasoning did not significantly predict ability to discriminate between true and fabricated stories. Both of these results are inconsistent with a generalised failure to think. Thus, it may be a mistake to infer that the more intuitive, reflexive cognitive style of endorsers shows that they are lazy and do not analyse (Pennycook and Rand 2019). Instead, performance on our more realistic test of analytical performance shows that endorsers may be *less* reflective or have *limited* analytical skills compared to non-endorsers. This interpretation is further supported by the observed differences in persuasiveness ratings between those who endorse implausible claims and those who do not.

Overall, endorsers were more persuaded by the presented evidence than non-endorsers. This general overvaluing could be because endorsers were relatively more optimistic about the strengths of evidence, and/or less pessimistic about the weaknesses of the evidence—although the former appears more likely given our data. Either way, the result suggests that endorsers differ from non-endorsers in their perceptions of what is or should be persuasive. Consequently, we may need to consider different strategies for reducing implausible belief formation and maintenance than those typically described in the literature.

Researchers examining implausible beliefs and cognitive style have tended to advocate for interventions that will shift people towards a more deliberative, reflective analytical strategy, for example, by ‘slowing down for a moment’ (Ward and Garety 2017, see also Bronstein et al. 2019; Greene and Murphy, this issue; Pennycook and Rand 2019). These suggestions are supported by experimental studies showing that implausible beliefs are reduced by additional deliberation time and information processing resources (Bago et al. 2020; Swami et al. 2014). Yet, it is unclear how much encouragement to deliberate would have changed the responses of endorsers in our sample. Instead, the generalised overvaluing of evidence suggests that endorsers may need help to appreciate the *impact* of various strengths and weaknesses on evidence quality. Thus, interventions focused on building analytical competence—for instance through education about research methods or threats to validity (McAuliff et al. 2009)—may be a promising avenue for further research.

### Limitations

It is important to be aware of some limitations when considering our results. First, we did not explicitly measure the cognitive style of our participants using, for example, the CRT or the AOT. As a result, we do not know whether endorsers in our sample had a more or less reflective analytical style than non-endorsers. We can only say that endorsers engaged in a reflective form of evidence evaluation that resulted in high-quality evidence being rated as more persuasive than low-quality evidence. Future research could measure both analytical performance and cognitive style to examine whether aspects of cognitive style can help to explain the differences between endorsers and non-endorsers that we observed.

It is also important to acknowledge that we used an ad hoc approach for assessing beliefs in implausible claims. We included three implausible claims in a general knowledge test battery and classified those who strongly believed any one of the claims as ‘endorsers’, and those who regarded all of them as more false than true as ‘non-endorsers’. This approach may have resulted in over- or under-inclusive definitions, which in turn could affect our results. However, the distribution of endorsement ratings suggests it is unlikely that the composition of endorsement groups would substantially change if we used more or less conservative definitions (see “Appendix A”). We also conducted post hoc analyses to examine the possible effects of different definitions on our results and found that both endorsers and non-endorsers were sensitive to evidence quality irrespective of the composition of non-/endorser groups or the evidence quality manipulations (see “Appendix C”). Nevertheless, it is important for future studies to replicate our findings using data collected primarily for that purpose.

## Conclusions

Overall, our study suggests that it is not laziness that separates those who believe implausible claims from those who do not. Instead, limited analytical skills may play a role in the development and maintenance of a range of implausible beliefs. These limitations could be addressed through interventions targeting evaluative performance. However, further research examining the relative contributions of cognitive style and analytical skill is vital for developing the most effective interventions to minimise the harms caused by implausible beliefs.

## Figures and Tables

**Fig. 2:**
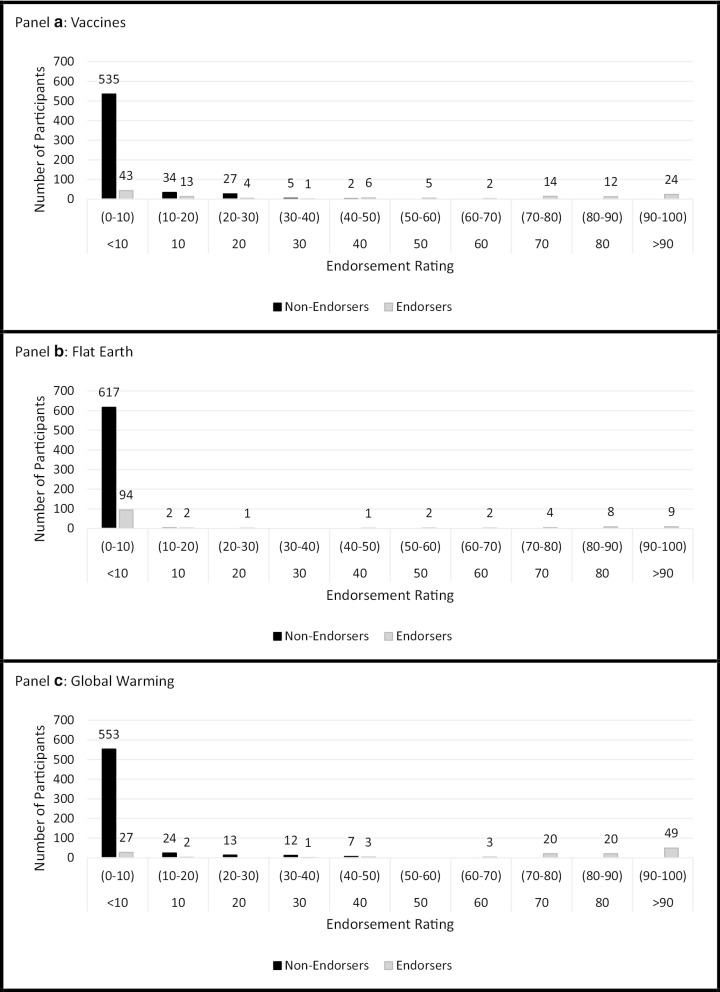
Distribution of endorsement ratings for vaccine, flat earth, and global warming claims by endorsement status

**Fig. 3:**
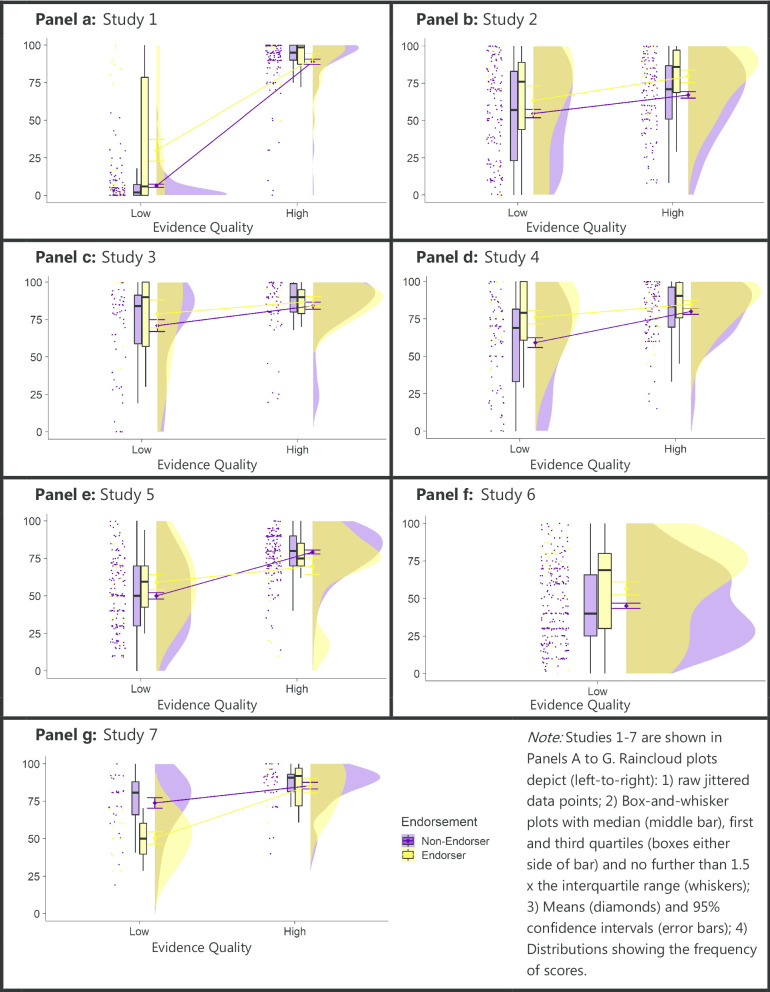
Summary of evidence persuasiveness by evidence quality and endorsement status in Studies 1–7

## Data Availability

The datasets generated and/or analysed during the current study are available in the Open Science Framework https://tinyurl.com/y4e75wo2.

## Appendix A

See Fig. 2.

## Appendix B

See Fig. 3.

## Appendix C

See Table 3.

**Table 3:** Post hoc linear mixed-effects models 1 and 2 predicting credibility, value and weight from evidence quality, endorsement status, and the quality by endorsement interaction

	Mean (SD)	*b*	SE	*t*	95% CI	Cohen’s *d*
*Model 1: All eligible participants (N* = *1747)*						
Evidence quality	–	30.00	1.56	19.25	26.94, 33.05	1.00
Low	51.6 (34.7)					
High	78.9 (21.9)					
Endorsement status	–	11.64	2.46	4.73	6.81, 16.46	0.24
Endorser	69.2 (30.8)					
Non-endorser	63.2 (32.7)					
Quality × Endorsement	–	− 13.67	3.85	3.55	− 21.19, − 6.12	0.18
Low/endorser	60.6 (34.8)					
Low/non-endorser	50.2 (34.4)					
High/endorser	78.9 (22.1)					
High/non-endorser	78.9 (21.8)					
*Model 2: ‘Endorsers’ any item* > *60; ‘Non-Endorsers’ all items* < *40*						
Evidence quality	–	32.66	2.42	13.51	27.95, 37.42	1.02
Low	49.4 (32.1)					
High	80.3 (20.4)					
Endorsement status	–	10.93	2.69	4.06	5.66, 16.20	0.29
Endorser	67.7 (29.6)					
Non-endorser	61.4 (32.0)					
Quality × Endorsement	–	− 11.60	4.67	2.48	− 20.73, − 2.43	0.18
Low/Endorser	57.9 (31.8)					
Low/Non-endorser	47.0 (31.8)					
High/Endorser	81.0 (19.6)					
High/Non-endorser	80.1 (20.6)					

## Abbreviations

AOT: Actively open-minded thinking
CRT: Cognitive reflection test
DNA: Deoxyribonucleic acid
MS2R: Motivated system 2 reasoning
NRC: National Research Council
PCAST: President’s Council of Advisors on Science and Technology
WHO: World Health Organization
